# Miscellaneous

**Published:** 1888-04

**Authors:** 


### MISCELLANEOUS.

THE MELBOURNE INTERNATIONAL EXHIBITION.—Australia celebrates the centenary of her settlement by a grand International Exhibition at Melbourne, commencing August 1st next, and continuing for six months. Congress has appropriated $50,000, will appoint commissioners to represent the United States, and it is hoped that very many private firms will make exhibits. The government of Victoria controls the exhibition and gives space and steam for motive power free. The exhibition building and annexes will cover twenty-four acres of ground, and the display, both local and from foreign countries, will be the largest ever seen in the southern hemisphere. Goods for exhibition are admitted duty free. Australia is a rich and growing country. American goods sell well there, and the comparative nearness of Australia to America should inspire our business men to control her markets. Mr. John M. Ives, who for the past three years has been in the antipodes as manager for the well known Safe Cure House of H. H. Warner & Co., has just returned with letters from the chief secretary and commissioners ; also blank applications for space, which he will be pleased to send free on application to him at Rochester, N. Y. Applications should be made at once, as goods must be in position not later than July first next.

PAIN masters the mind. Avoid the disease known as Catarrh, buy using Warner's Log Cabin Rose Cream. 50c. Sold by all druggists.

TO REMOVE A CINDER FROM THE EYE.—A few years since, I was riding on the engine of the fast express, from Binghamton to Corning. The engineer, an old schoolmate of mine, threw open the front window and I caught a cinder that gave me the most excruciating pain. I began to rub the eye with both hands. "Let your eye alone and rub your other eye ;" this from the engineer. I thought he was chaffing me, and worked the harder. "I know you doctors think you know it all, but if you will let that eye alone, the cinder will be out in two minutes," persisted the engineer. I began to rub the other eye, and soon I felt the cinder down near the inner canthus, and made ready to take it out. "Let it alone, and keep at the well eye," shouted the doctor *pro-tem.* I did so for a minute longer, and looking in a small glass he gave me, I found the offender on my cheek. Since then I have tried it many times, and have advised many others, and I never have known it to fail in one instance (unless it was as sharp as a piece of steel, or something that cut into the ball and required an operation to remove it.) Why it is so I do not know. But that it *is* so I do know, and that one may be saved much suffering if they will let the injured eye alone and rub the well eye. Try it.—DR. R. W. ST. CLAIR in *Medical Summary.*

Cases of actual heart disease are rare. Cases in which the heart is affected sympathetically through some derangement of stomach, bowels or uterus are common. A woman was treated for years for "heart disease," and lived all that time in the awful suspense of expecting to drop dead any moment. A wiser physician discovered that the disturbance felt in the heart was due to an affection of the womb. This was promptly cured and nothing further was heard of the heart trouble.

The importance of keeping the feet warm and the head cool cannot be over estimated. If such a condition was always maintained, congestions would be rare. The tendency of heat is upward, and, as a consequence, the temperature is higher at the ceiling than at the floor. This is particularly true of rooms heated by hot air furnaces. It is a condition antagonistic to health. A simple device has recently been patented for arresting in its upward course the heat from a furnace register and throwing it out upon the floor.

Bad Government demoralizes its subjects. Put the demoralized stomach in proper order by the use of Warner's Log Cabin Hops and Buchu Remedy. Largest bottle proprietary medicine in the market. Try it. Sold by all druggists. Take no other hops and buchu.

### A DEAR LITTLE MAID.

Right over against my window,
Now, that the days are long,
A dear little maid
Oft sits in the shade,
And tuneth her words to song.

She singeth of sorrowful things,—
Of gallants that never were true,
And methinks as I list;
Little maiden, I wist
The dream that troubleth you.

Athene! bright goddess of home,
And graces that ever delight,
O! printhee, give heed,
Where love hath such need,
Of wisdom to guide it aright.

Why stand ye aloof, whensoever
The dear little mischievous elf,
His arrows lets go;
Come weal or come woe,
But hideth away himself!

It were not amiss, if together,
Ye gave the swift message aim,
That reason be made,
By passion unswayed,
And love never kindled in vain.

H. De T.

